# Whole slide images as non-fungible tokens: A decentralized approach to secure, scalable data storage and access

**DOI:** 10.1016/j.jpi.2023.100350

**Published:** 2023-11-09

**Authors:** Arlen Brickman, Yigit Baykara, Miguel Carabaño, Sean M. Hacking

**Affiliations:** a Department of Pathology and Laboratory Medicine, Warren Alpert Medical School of Brown University, Providence, RI, United States; b Department of Pathology, NYU Langone Health, NYU Grossman School of Medicine, New York, NY, United States

**Keywords:** WSI, NFT, Data storage, Data transfer, Decentralization, DLT, Blockchain, Technical report

## Abstract

**Background:**

Distributed ledger technology (DLT) enables the creation of tamper-resistant, decentralized, and secure digital ledgers. A non-fungible token (NFT) represents a record on-chain associated with a digital or physical asset, such as a whole-slide image (WSI). The InterPlanetary File System (IPFS) represents an off-chain network, hypermedia, and file sharing peer-to-peer protocol for storing and sharing data in a distributed file system. Today, we need cheaper, more efficient, highly scalable, and transparent solutions for WSI data storage and access of medical records and medical imaging data.

**Methods:**

WSIs were created from non-human tissues and H&E-stained sections were scanned on a Philips Ultrafast WSI scanner at 40× magnification objective lens (1 μm/pixel). TIFF images were stored on IPFS, while NFTs were minted on the Ethereum blockchain network in ERC-1155 standard. WSI-NFTs were stored on MetaMask and OpenSea was used to display the WSI-NFT collection. Filebase storage application programing interface (API) were used to create dedicated gateways and content delivery networks (CDN).

**Results:**

A total of 10 WSI-NFTs were minted on the Ethereum blockchain network, found on our collection “Whole Slide Images as Non-fungible Tokens Project” on Open Sea: https://opensea.io/collection/untitled-collection-126765644. WSI TIFF files ranged in size from 1.6 to 2.2 GB and were stored on IPFS and pinned on 3 separate nodes. Under optimal conditions, and using a dedicated CDN, WSI reached retrieved at speeds of over 10 mb/s, however, download speeds and WSI retrieval times varied significantly depending on the file and gateway used. Overall, the public IPFS gateway resulted in variably poorer WSI download retrieval performance compared to gateways provided by Filebase storage API.

**Conclusion:**

Whole-slide images, as the most complex and substantial data files in healthcare, demand innovative solutions. In this technical report, we identify pitfalls in IPFS, and demonstrate proof-of-concept using a 3-layer architecture for scalable, decentralized storage, and access. Optimized through dedicated gateways and CDNs, which can be effectively applied to all medical data and imaging modalities across the healthcare sector. DLT and off-chain network solutions present numerous opportunities for advancements in clinical care, education, and research. Such approaches uphold the principles of equitable healthcare data ownership, security, and democratization, and are poised to drive significant innovation.

## Introduction

The digitalization of healthcare has led to an increased flow of data, resulting in a pressing need for privacy-preserving technologies that can improve research and clinical applications within the healthcare ecosystem. We will focus on digital pathology for the proof-of-concept as it is revolutionizing the way pathology is practiced, improving diagnosis, treatment, and research. Hospital systems are increasingly adopting digital pathology, which involves digitizing glass slides into virtual whole-slide images (WSI) and using computer technology to analyze and interpret the resulting images.^1^ Whole-slide images represent the most complex and sizable data files in healthcare. Successfully employing our proposed method for these images suggests its applicability to other, less-dimensionally complex data and imaging modalities across the medical field. Efficient storage, retrieval, and sharing of patient data enables swifter and more precise diagnoses, ultimately improving patient outcomes and care. WSIs necessitate high-resolution storage, while the costs associated with local and cloud-based storage solutions present a major barrier to the widespread adoption of digital pathology.^2^

Distributed ledger technology (DLT), or “Blockchain”,^3^ coincides with off-chain network storage solutions, which may hold the key to unlocking the full potential of medical records, medical imaging, and digital pathology. Blockchain is a first-generation DLT, introduced by Satoshi Nakamoto as Bitcoin.^4^ Blockchain comprises a growing list of records known as blocks. Each block contains a timestamp, transaction data, and a unique hash value that connects it to the previous block, which creates an immutable chain of blocks (on-chain) that ensures the integrity and security of the data.^5^

On the other hand, off-chain network storage solutions are decentralized methods of storing data that often utilize unused hard disk space of users across different nodes to store files.^6^ Such protocols are expected to be less expensive than centralized cloud storage. The InterPlanetary File System (IPFS) is an off-chain protocol skillfully amalgamating the realms of hypermedia and peer-to-peer file-sharing networks to facilitate distributed data storage and dissemination. WSIs inherently possess immense size dimensionality, and IPFS presents itself as a possible solution to address the unique challenges posed by the extensive size constraints of non-fungible tokens (NFTs) minted on-chain. IPFS employs Content Identifiers (CIDs) as a mechanism for efficient storage and transmission in a compressed binary format.

Filebase, a user-friendly cloud storage platform, leverages decentralized networks like Filecoin for secure and affordable storage, offering a simple interface for users to benefit from decentralized storage without blockchain complexities, and are well-suited for digital pathology storage applications. MetaMask, a non-custodial NFT wallet, stores private keys on users' devices instead of a centralized database. OpenSea, a leading decentralized NFT marketplace launched in 2017, showcases a wide variety of digital assets, making it a suitable platform for hosting and sharing our WSI collection.

The objective of this study is 3-fold: firstly, to exhibit a architecture that delineates actual WSI storage from metadata and data persistence, secondly, to demonstrate the utility of a non-custodial cryptocurrency wallet, MetaMask, which can be used to manage WSI-NFTs and interact with Ethereum-based decentralized applications (dApps), including OpenSea. Thirdly, we aim to demonstrate the utility of Filebase storage application programing interface (API) for improved WSI data access and content delivery. By executing these goals, this technical report seeks to illuminate the possibilities that DLT and off-chain network storage solutions offer in the context of digital pathology and for the management of WSIs. These solutions could improve performance, security, and transparency in healthcare, while also empowering patients as the rightful owners of their medical data.

## Methods

### Whole-slide images

Digital WSIs were generated utilizing non-human tissue samples, with the primary inclusion criterion being the presence of adequate viable tissue for staining and scanning procedures. Hematoxylin and Eosin (H&E) stained sections were meticulously scanned employing a Philips Ultrafast WSI scanner, equipped with a 40× magnification objective lens, yielding a resolution of 1 μm per pixel. Subsequently, all digital slides were procured from the vendor-agnostic whole-slide image viewer in a Pyramidal Tag Image File Format (TIFF) image format, accompanied by variable degrees of Joint Photographic Experts Group (JPEG) image compression. Given the exclusive utilization of non-human tissue samples, the requirement for Institutional Review Board (IRB) approval was obviated. Here, we proposed a 3-layer architecture for scalable WSI data storage and access based on Ethereum ERC-1155 standard, IPFS along with Filebase API for enhanced content delivery and retrieval due to the size complexity of WSIs.

### Ethereum non-fungible tokens (Layer 1)

**Fig. 1:**
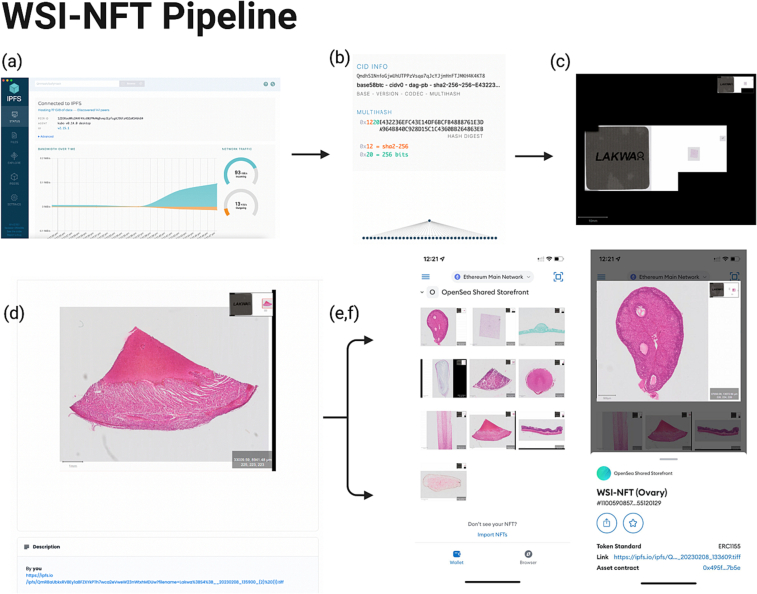
Pipeline for storing, minting, accessing, and transferring ownership of WSI-NFTs. (a) TIFF WSI Files being stored using IPFS on IPFS desktop, (b) viewing IPFS content including CIDv0 on IPFS gateway, (c) Qupath being used to view TIFF-WSI downloaded from IPFS, and (d) IPFS metadata was included being included under the description section of the OpenSea webpage for easy access. MetaMask allowing for viewing of tokens natively in the user interface with access to the IPFS CID link, along with information of token standard, asset contract link, and the ability to transfer ownership on the Ethereum main network.

NFTs were minted on Ethereum blockchain via API provided by https://opensea.io. All NFT metadata was structured according to ERC-1155 standard (Ethereum Request for Comments 1155), a novel token standard leverages the best from previous standards to create a fungibility-agnostic and gas-efficient token contract, with both fungible and non-fungible tokens within a single smart contract. ERC-1155 represent a NFT Standard that implements an API for tokens within Smart Contracts. IPFS metadata was included as described above, and the IPFS CID was included under the description section of the OpenSea webpage (Fig. 1d). MetaMark allows users to manage, store and transfer NFTs in their MetaMask wallet when minted on OpenSea, importing the NFT simply allows the user to see the token natively in the user interface (UI) (Fig. 1e–f). This is precisely why users can only add NFTs which they own to their MetaMask. OpenSea does not store NFTs, however as an Ethereum-based dApp it does allow users to manage WSI-NFTs from their respective wallets. An overview WSI-NFT pipeline and study overview is available in Fig. 1.

### Decentralized storage on IPFS (Layer 2)

TIFF WSI Files were stored using IPFS on IPFS desktop (kubo v0.14.0 desktop), (Fig. 1a). IPFS is a wonderful solution for storing and addressing data dimensionality in the NFT space, particularly as most NFTs minted on the blockchain are limited to 50–100 MB in size. IN IPFS, CIDs uniquely identify a piece of content. A CID can be stored and sent over the network in a compact binary form, but they're represented as strings of random-seeming characters when displayed to users.

For this study, we opted for using CIDv0 “version 0” instead over the newer CIDv1 "version 1" as it is more simple, however, not always as versatile as the newest CID version. However, CIDv0 is the default version most IPFS applications and operations and it is recommended that applications support CIDv0 in any development environment. CIDv0 CIDs are 46 characters long and always start with the characters “Qm” (as shown in Fig. 1b). Once we received the CID for the WSIs added to IPFS, we proceeded to prepare the token's metadata and "mint" the token on an Ethereum blockchain. To link the content from a smart contract within the NFT's metadata, we converted the CIDv0 to an IPFS URI, which is the canonical representation of an IPFS link. The use of the static string "ipfs://." ensures that the CID is unambiguously associated with content on IPFS rather than any other system.

TIFF WSIs downloaded from IPFS can be visualized on any TIFF image viewer that supports the TIFF format. This includes browser extensions that can display TIFF images, or open-source WSI viewer solutions such as Qupath^7^ (Fig. 1c). To view TIFF images downloaded from IPFS, you can simply open them with any TIFF viewer installed. Alternatively, you can use a browser extension that supports the TIFF format to view the images directly in your browser. It's important to note that the performance of viewing TIFF images may vary depending on the size and complexity of the images and the capabilities of the viewer or browser extension being used.

### Filebase storage API (Layer 3)

Filebase is the premier S3-compatible object storage platform, enabling secure, redundant, and high-performance data storage across various decentralized storage networks. In this report, all 10 WSIs were re-pinned via CIDs on Filebase (https://filebase.com/), this allows the WSI data to be saved and announced from 3 different data centers around the world. The net result is more IPFS peers announcing the content, which greatly improves retrievability and performance. In addition to the above, these dedicated gateways provided allow for the creation of a HTTP link that is served by a content delivery network (CDN), resulting in even greater retrieval performance.

## Results

10 WSIs were minted on the Ethereum chain with unique histology created from non-human tissue cases arising from surgical biopsy and resection material. WSI tissue included ovary, zea root, spinal cord, testis, zea seed, leaf of nerium indicum, bacteria, adipose tissue, rectum, and heart. Under optimal conditions, WSIs were found to be able to be retrieved at speeds of over 10 mb/s, however, download speeds and WSI retrieval times varied significantly depending on the file and gateway used. Files retrieved from dedication gateways and CDN provided by Filebase API resulted in faster and more consistent download speeds when compared to downloaded from the public gateway via IPFS desktop (Table 1).

**Table 1:** Overview of WSI-NFTs with associated characteristics and associated CID.

NFT	Chain	Links	Size	1. CID (with https link) 2. Filebase dedicated gateway (CDN)
Ovary	Eth	39	1.6GB	https://ipfs.io/ipfs/QmSsP2Q11BWwEsMpp3T3g1HhFepbFLaSxJkt6Eivb5GtDj?filename=Lakwa%3BS10%3B__20230208_133609.tiff https://wsi-nft.s3.filebase.com/1
Zea Root	Eth	38	1.7GB	https://ipfs.io/ipfs/QmV1EFbry6oDrkBcfnLBwr52ojRicq7UGxR7PN491QLKHd?filename=Lakwa%3BS14%3B__20230208_132308.tiff https://wsi-nft.s3.filebase.com/2
Spinal cord	Eth	38	1.7GB	https://ipfs.io/ipfs/QmWnz1EfHS83FUetDB5PPZxkcc8zEwEbMfL5YeQJ94VF5g?filename=Lakwa%3BS13%3B__20230208_132551.tiff https://wsi-nft.s3.filebase.com/3
Testis	Eth	48	2.2GB	https://ipfs.io/ipfs/QmdpWjvtAwg415ePqZZUR5mrm6cPKJEG1K8XWb3wnJSTbq?filename=Lakwa%3BS12%3B__20230208_133015.tiff https://wsi-nft.s3.filebase.com/4
Zea seed	Eth	41	1.9GB	https://ipfs.io/ipfs/QmbLyPR1EzUa7YncEkWEejDg1aeWFfnyAMti84YrZkWWvA?filename=Lakwa%3BS21%3B__20230208_161204.tiff https://wsi-nft.s3.filebase.com/5
Leaf of nerium indicum	Eth	41	1.9GB	https://ipfs.io/ipfs/QmcEehBRjPPVsX6fwWH9tE5WPj1wsRQ6CyE9xsRaruhTsF?filename=Lakwa%3BS20%3B__20230208_125847.tiff https://wsi-nft.s3.filebase.com/6
Bacteria	Eth	37	1.6GB	https://ipfs.io/ipfs/QmdhS1NnfoGjwUhUTPPzVsqa7qJcYJjmHnFTJMKH4K4KT8?filename=Lakwa%3BS2%3B__20230208_140555.tiff https://wsi-nft.s3.filebase.com/7
Adipose tissue	Eth	39	1.7GB	https://ipfs.io/ipfs/QmRYZSoGfyJrQdbVK9LbSLxVy3zhjYLuJaArNA2ZzLrcuX?filename=Lakwa%3BS26%3B__20230208_155827_(2).tiff https://wsi-nft.s3.filebase.com/8
Rectum	Eth	37	1.7GB	https://ipfs.io/ipfs/QmdV74ws97tdzEyhpyiMWc8JcYpGEZoG2qUMJtahdusSg1?filename=Lakwa%3BS6%3B__20230208_135135_(2).tiff https://wsi-nft.s3.filebase.com/9
Heart	Eth	44	2.0GB	https://ipfs.io/ipfs/QmRBaUbkxRVBEy1aBFZXYkPTh7wca2eVweW23nWtxhMDUw?filename=Lakwa%3BS4%3B__20230208_135900_(2)%20(1).tiff https://wsi-nft.s3.filebase.com/10

NFT, Non-fungible token; Eth, Ethereum; CID, Content identifier, CDN, Content delivery network.

Any delayed retrieval times on the IPFS public gateway are likely a result of the large size content and low number of hosting nodes. The larger the file, the more blocks that need to be found, and the less likely that it will be able to track down all the pieces. Previous experimental studies have revealed that both resolving and downloading operations can become bottlenecks in optimizing IPFS in avoiding high-latency I/O (input/output) operations.^8^

### Non-fungible tokens

10 NFTs were showcased with IPFS download links on OpenSea: https://opensea.io/collection/untitled-collection-126765644. All NFT metadata was structured according to ERC-1155 standard. NFTs were stored on a MetaMask wallet (*Version 10.25.0*) at the following public address: 0xF3533C29206B269a46613d9E0EDD8516A5630aC7. An overview of the 10 NFTs which were minted with characteristics is available in Table 1. Click on the CID (IPFS public gateway) or Filebase gateway with https link to download and access the WSIs yourself. Overall, we found the Filebase dedicated gateways with HTTP link to result in greater data retrieval performance.

## Discussion

NFTs have emerged as a promising management solution, addressing the governance of data at both personal and institutional levels.^9^ This burgeoning demand is propelled by the escalating digital flux of data and the imperative for privacy-preserving technologies that augment research and clinical applications in healthcare.^9^, ^10^, ^11^

DLT and off-chain network solutions now possess the potential to unleash the untapped capabilities of WSIs. Our innovative approach incorporates IPFS, alleviating storage constraints on layer 1 DLTs, such as Ethereum, and paving the way for a more efficient digital pathology landscape. The principal controlling file for these applications will be the JSON file, which would interact with the smart contract infrastructure and assume responsibility for token minting.

Subramanian et al.^12^ previously employed the smart contract NFT standard (ERC-721) to mint WSI-NFTs on the Ethereum network, utilizing the JSON file's IPFS URL to store WSIs. Using a 2-layer architecture, ownership details were documented in the metadata file, while access implementation was facilitated through functions supported by the ERC-721 standard. Such methodologies enable the segregation of ownership and access properties, rendering them ideally suited for the realm of digital pathology and medical data.

In our approach, we used the ERC-1155 standard, which allows for the creation of both fungible and non-fungible tokens within 1 contract, unlike ERC-721, which is designed for unique NFTs and requires a separate transaction for each token transfer. The choice between ERC-721, ERC-1155, or other token standards depends on a project's needs. ERC-721 is suitable for unique WSI representation, while ERC-1155 is preferable for cases requiring both unique and interchangeable tokens or optimized efficiency and cost, such as projects with multiple WSIs.

The use of NFTs and DLT for WSIs is still in its early stages and there are limitations to consider, such as the potential lack of interoperability across different blockchain platforms and the potential for increased complexity in data management. Furthermore, the use of NFTs raises questions about data ownership and access. While NFTs allow for the separation of ownership and access properties, as discussed in this study, the issue of who owns the data and how it can be accessed remains a complex issue that requires further exploration. Despite these challenges, the potential benefits of utilizing NFTs and DLT for WSIs are significant. By balancing the potential benefits and challenges associated with these technologies, we can ensure that they are used in a way that maximizes their potential to improve healthcare outcomes while mitigating any potential risks.

It is crucial to highlight the potential for integrating our approach with decentralized artificial intelligence (AI)-based methodologies such as swarm learning (SL),^13^ a technique that enables partners to collaboratively train AI models without necessitating data transfer or monopolistic data governance. SL and decentralized-AI utilizes the blockchain to securely store and manage model updates, ensuring equitable contributions from each data source during the training process.

Saldanha et al.^14^ successfully demonstrated the efficacy of SL in extensive datasets encompassing over 5000 patients, revealing that AI models trained using SL could predict *BRAF* mutational status and microsatellite instability directly from routine pathology slides. The SL-trained AI models consistently outperformed locally trained models, exhibiting data efficiency. This study indicates that SL can be employed to train distributed AI models for any histopathology image analysis task, effectively eliminating the need for data transfer and paving the way for more secure, decentralized approaches to digital pathology. In future, such AI technologies could live on the blockchain, enabling a more inclusive and fair sharing of machine learning model weights, while also democratizing patient access to AI technology.

Subsequent research efforts should be directed towards developing more optimized WSI-NFT and metadata standards, particularly considering the swiftly evolving technological landscape.^15^ Establishing such standards would signify a significant advancement in the application of NFTs for ownership and management of large-scale image data. Such a standard could promote more optimized precision medicine API, interoperability, and foster the secure, ethical utilization of WSIs. Although more nuanced models for tailoring NFT metadata may be needed to serve different use cases.^16^

It is worth considering the potential limitations and challenges associated with the use of NFTs and DLT for WSIs. One potential concern is the scalability of some layer 1 blockchain technologies such as Bitcoin, which cannot scale to high transactions. The storage and transfer of large volumes of data, such as WSIs, could also be difficult to handle within the current limitations of blockchain technology. Additionally, the use of DLT requires significant technical expertise, which may limit its adoption by clinicians, researchers, and other professionals.

We also observed that using a dedicated gateway served by a CDN, as seen with our use of Filebase API, resulted in greater retrieval performance, compared to our public IPFS gateway. Poor performance with IPFS can be attributed to various factors, including the intermittent online status of many nodes, and the selective storage behavior of nodes. Link rot in IPFS occurs due to reasons such as transient nodes, lack of data replication, data pinning practices, network partitioning, content updates or deletion, and resource limitations. Transient nodes are often not online 24/7, making content temporarily or permanently unavailable. IPFS relies on data replication for content availability, and a lack of replication can also make content inaccessible. Data pinning allows nodes to retain specific data, however WSIs unpinned on IPFS can be lost over time.

This technical report proposes a 3-layer architecture for scalable WSI data storage and access based on Ethereum for minting, IPFS for decentralized information storage and transmission, and finally Filebase API for dedicated gateways and content delivery. NFTs are poised as a solution for managing personal and institutional data governance, driven by the increased digital flow of data and the need for privacy-preserving technologies that improve research and clinical applications within our healthcare ecosystems. Such development represents an important step forward in the digital transformation of healthcare. Finally, for those interested in contributing to the longevity of this project, we encourage you to pin our WSI-NFT collection using IPFS.

## Declaration of Generative AI and AI-assisted technologies in the writing process

During the preparation of this work the authors used GPT-4 (https://chat.openai.com/) to improve readability and language. After using GPT-4, the authors reviewed and edited the content as needed and take full responsibility for the content of the publication.

## Declaration of Competing Interest

Author SH has equity ownership in Odyssey HealthCare Solutions Inc. The remaining authors have no remaining possible conflicts of interest to disclose.

## Data Availability

All data and WSI collected and stored in the present study can found and downloaded at https://opensea.io/collection/untitled-collection-126765644.
